# 3.F. Round table: Better DiPH – To plan, implement, evaluate, and the future of digital public health interventions

**DOI:** 10.1093/eurpub/ckac129.147

**Published:** 2022-10-25

**Authors:** Organised by: Leibniz ScienceCampus Digital Public Health Bremen LSC (Germany), EUPHA-DH, Chair persons: Laura Maass (Germany), Chen-Chia Pan (Germany)

## Abstract

The potential of digital technology for improving the health of individuals, communities, and populations is unprecedented. Technological advancements empower individuals to engage in self-monitoring and self-management of their chronic conditions or health and well-being. There is an unparalleled opportunity to reform prevention, health promotion, and healthcare services with lower cost and better reach and accessibility. However, health technologies are often developed without supportive evidence or a user-centred design. This leads to a lack of long-term user engagement in digital public health interventions. Our workshop aims to facilitate a mutual understanding of the specific properties of digital public health tools by creating a space for discussing the various perspectives of such technologies. We want to start a conversation of essential steps for conceptualising, implementing, and evaluating needs-based and society-centred digital public health interventions to improve the acceptability and sustainability of such interventions in users. The workshop will address digital public health tools on different steps and describe the progression as an iterative approach to highlight where these aspects are linked. The first speaker will provide a theory-guided overview of digitalisation in health to create a shared understanding of the terminology for the workshop. This includes the differentiation between digital health and digital public health. The talk will highlight the importance of digital tools for surveillance, monitoring, healthcare, health promotion, and their significant meaning for society. Following this input, the other panellists will guide us through different aspects of digital public health tools: The second speaker will discuss the importance of society-centred designs based on users’ needs rather than on technological advancements for interventions. Our third speaker will present a meta-framework of extended criteria for developing and evaluating digital technologies for public health. The fourth panellist will share Malta's COVID-19 contact tracing app as a case study. He will discuss the challenges and facilitators in implementing and evaluating digital public health interventions. The last presentation will cover the need for governmental support in the future to ensure the success of digital public health interventions and holistic systems. The workshop will take place as a round table discussion. Each panellist will give a short (7 minute) input talk on the specific properties of digital public health tools. After the panellists present their opinion, we will open the floor for a discussion. Here, the audience is invited to share their knowledge and experiences to build a mutual understanding of the crucial steps in digital public health interventions. After the workshop, we will create a white paper on digital public health based on the panellists’ input and the discussion results.

**Key messages:**

• A mutual understanding of digital public health may facilitate public sector cooperation and aim towards needs-based and society-centred technology development to improve the population's health.

• Digital public health offers unique challenges, and there is an opportunity to outline these specific nuances to ensure maximum success in implementing such projects.

